# Deep Neural Network-Based Cigarette Filter Defect Detection System with FPGA Acceleration for Online Recognition

**DOI:** 10.3390/s24206752

**Published:** 2024-10-21

**Authors:** Liang Huang, Qiongxia Shen, Chao Jiang, You Yang

**Affiliations:** 1 School of Electronic Information and Communications, Huazhong University of Science & Technology, Wuhan 430074, China; d202080755@hust.edu.cn; 2 Fiberhome Telecommunication Technologies Co., Ltd., Wuhan 430205, China; qxshen@fiberhome.com (Q.S.); jiangchao@fiberhome.com (C.J.)

**Keywords:** defect detection, deep neural network, field programmable gate array, real-time

## Abstract

In the cigarette manufacturing industry, machine vision and artificial intelligence algorithms have been employed to improve production efficiency by detecting product defects. However, achieving both high accuracy and real-time defect detection for cigarettes with complex patterns remains a challenge. To address these issues, this study proposes a model based on RESNET18, combined with a feature enhancement algorithm, to improve detection accuracy. Additionally, a method is designed to deploy the model on a field-programmable gate array (FPGA) with high parallel processing capabilities to achieve high-speed detection. Experimental results demonstrate that the proposed detection model achieves a detection accuracy of 95.88% on a cigarette filter defect dataset with an end-to-end detection speed of only 9.38 ms.

## 1. Introduction

In some cigarette factories, some high-end cigarettes have unique patterns designed on the filter to enhance the visual appeal of the product. However, this poses challenges for defect detection on the production line. Simple patterns can be easily detected using traditional methods such as pixel comparison and template matching. However, defects in complex patterns are difficult to identify even for the human eye, and they can easily be confused with the background, making it even more challenging for machines to accurately detect them. Using deep neural networks (DNN) to detect such defects has become a necessary consideration.

Recently, with the rapid development in the deep learning field, defect detection methods based on DNN have been used in various production fields. Among these applications, there are also products with complex patterns and backgrounds. Compared with traditional vision methods, DNN methods offer significant advantages in terms of development efficiency and detection capabilities by learning and extracting features autonomously [1,2,3,4,5]. For example, a study [6] designed a DNN system for automatic identification of surface defects in metal manufacturing by leveraging the synergy of a DNN and transfer learning to improve defect detection accuracy and reinforce quality control on the production line. In addition, in the pharmaceutical sector, a previous study [7] described the application of a DNN that merges an autoencoder with a CNN to inspect tablets using a substantial dataset comprising both flawless and flawed samples. The outcomes confirmed the model’s ability to pinpoint diverse types of defects effectively. Some researchers [8] have discussed the application of DNNs for the visual inspection of printed circuit boards (PCB), where a CNN framework was utilized to pinpoint and categorize PCB defects, thereby improving accuracy and streamlining the quality inspection within the manufacturing workflow. For bottling production lines, a previous study [9] proposed a deep learning technique that integrates CNNs with recurrent neural networks to identify discrepancies, e.g., label misalignment and cap damage, and the evaluation results exhibited high precision and efficiency. For powdered component manufacturing, one study [10] introduced a DNN method that employs deep CNNs to classify prevalent defects, and the result highlighted the versatility of DNNs in advancing quality management in the powder industry.

In the cigarette industry, there have been several studies addressing defect detection in cigarette appearance. One study [11] proposed a C-CenterNet-based method that achieved an impressive 95.01% detection accuracy in detecting defects in cigarette appearance. In another study [12], various image processing techniques such as image smoothing, edge detection, binarization, and feature extraction were applied to the cross-sectional image of the filter rod, enabling the analysis of regions of interest and extraction of the number of filter rods. Similarly, image segmentation and morphological operations were utilized in a study [13] to distinguish cigarette regions from the background. A trilinear model was then employed to locate each cigarette and determine the presence of defects by calculating the number of pixels in the obtained regions. Other approaches, such as a self-learning control system based on a data acquisition card [14], can detect surface defects of cigarettes using a combination of databases, data acquisition technology, and industrial computers. Additionally, the use of the Canny operator for cigarette extraction and analysis of incomplete grayscale image regions was explored in a study [15]. Similarly, techniques like maximum contour area determination and template matching were used to identify both significant and subtle defects in cigarette appearance. However, these conventional methods may have limited adaptability to complex scenarios and often require manual intervention, which may result in limited generalization and lower detection accuracy, particularly for defects with indistinct edges, complex backgrounds, or non-flat surfaces.

**Figure 1:**
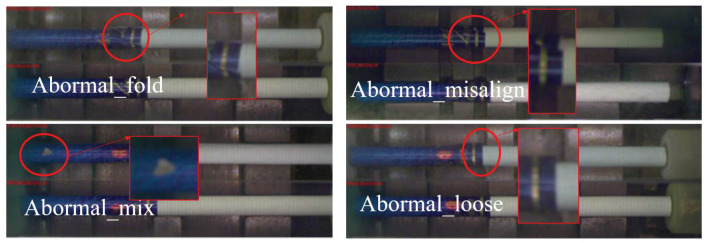
Cigarette filters with defects.

In the detection of cigarette products with complex-patterned filters, the style of the patterns greatly complicates the detection of defects. As shown in Figure 1, grid-like patterns can easily be confused with wrinkling defects, while spots in the pattern can be mistaken for specks of debris that have adhered to the cigarette. Additionally, as the cigarettes rotate on the production line, there is no fixed side that consistently faces the inspection system. Moreover, environmental factors in a production setting, such as dust, debris, and mist, can further interfere with the detection process. Therefore, this type of detection is more challenging than traditional defect detection. Aside from the requirements of the detection algorithms themselves, there is also a need for portability and flexibility in the hardware that carries these algorithms as detection may need to be deployed at various locations along the production line. In cigarette factories, there are dozens of production lines that manufacture products, with each line requiring detection at more than ten points. Some of these detection locations are quite cramped. Conventional PC + GPU setups, due to their larger size, are not suitable for some spaces where size and power consumption are limited. Additionally, because the detection results are needed to control the production line for defect product rejection, there are considerable requirements for the end-to-end latency of the detection. If the detection process is too slow, or if the transmission speed is too slow, it could result in defective products flowing into the next phase and being difficult to process by the system in a timely manner. In the factory where we conducted our trials, each batch consists of 200 cigarettes. The production line for the cigarettes, from manufacture to packaging, is about 2 m long, with a transfer speed of 1 m/s. Therefore, it is necessary to complete the detection and feedback results of each cigarette within 10 ms.

To address the aforementioned issues, this study has made efforts in both detection algorithms and hardware deployment. By developing a specialized DNN algorithm, we achieve precise detection and identification of complex texture defects. In addition, to satisfy the requirement for high-speed defect detection, we designed a DNN accelerator built on field programmable gate arrays (FPGA) technology to ensure efficient algorithm execution. The primary contributions of this study are summarized as follows:

- We proposed a DNN model that leverages the ResNet architecture and a feature enhancement module to improve the model’s ability to recognize complex texture cigarette defects.
- An inference acceleration solution based on Xilinx FPGA (Xilinx, San Jose, CA, USA) technology has been designed, allowing for the convenient deployment of the detection algorithm on the system, and the hardware itself can be installed at any location on the production line. Performance evaluations indicate that the FPGA accelerator surpasses traditional solutions in metrics of latency and efficiency.
- An integrated end-to-end defect detection solution is proposed, which encompasses image data preprocessing, detection, and feedback of results, all within a time frame of less than 10 ms, thus meeting the requirements for rapid detection and timely control.

The rest of this paper is organized as follows. Section 2 introduces the cigarette defect detection model, which includes the training process and inference performance. Hardware design based on FPGA, which deploys the proposed model, are shown in Section 3. Section 4 concludes the paper.

## 2. Model and Method

### 2.1. Cigarette Defect Dataset

Cigarettes are divided into filter and rod sections. For defects in the rod section, traditional visual processing methods can complete detection and removal tasks efficiently and accurately. However, the results are unsatisfactory for defects in the filter area, which frequently has a complex texture. Thus, the deep learning-based defect detection method used in our experiment targets the complex textures of the cigarette filter. Several common defects are shown in Figure 1. Here, tipping paper misalignment refers to when the filter paper sticker is not aligned during the production process, tipping paper wrinkling refers to wrinkling on the surface of the tipping paper caused by compression during the production process, tipping paper loosening refers to when the tipping paper does not adhere firmly to the cigarette during production, and tipping paper contamination refers to tobacco foam sticking to the surface of the tipping paper during production. It is very difficult to distinguish these defects due to the extremely complex texture of the tipping paper surface.

The experimental dataset was collected from the production line of a factory and contains 20,000 images with approximately equal numbers of images for each defect type. For each image, the corresponding image names were set as labels based on the different defects. Here, images of tipping paper misalignment are labeled “abnormal_align”, images of tipping paper wrinkling are labeled “abnormal_fold”, images of tipping paper loosening are labeled “abnormal_loose”, images of tipping paper contamination are labeled “abnormal_mix”, and defect-free images are labeled “normal”. After all of the images were labeled, the dataset was divided randomly into a training set and a testing set at a ratio of 7:3.

Considering the accuracy and speed requirements in online detection, it is necessary to minimize computational complexity while ensuring that key information is not lost. Thus, appropriate dataset preprocessing is crucial. The original image size of the dataset was 1100 × 320 pixels. In this study, the focus of recognition is the filter section of the cigarette; thus, the original images were cropped to retain only the left side of the image with the filter, effectively reducing the image size to 512 × 320 pixels. In order to be compatible with the input size of commonly used neural network models, the images were resized to a resolution of 224 × 224 pixels. Next, the images were normalized to further optimize the uniformity of the dataset.

### 2.2. Baseline Model Selection

**Figure 2:**
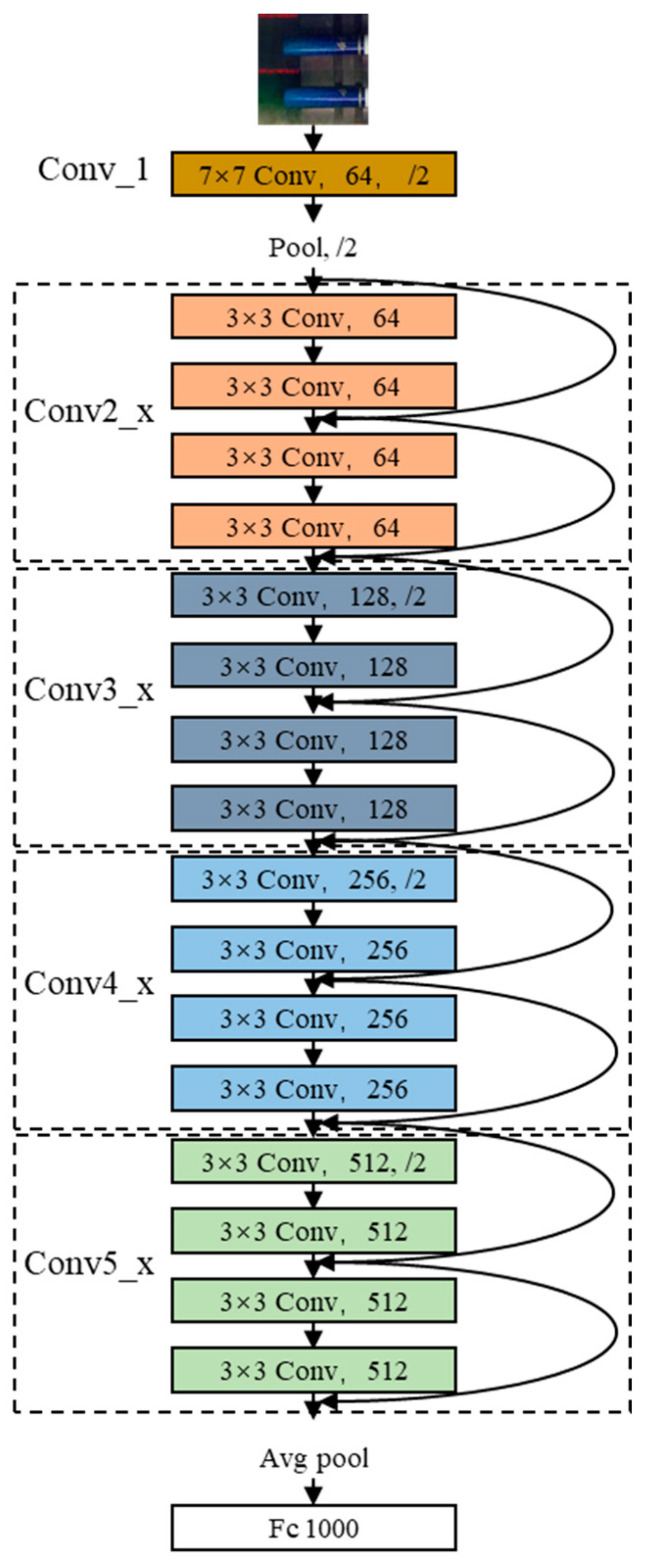
Structure of ResNet18.

The defect detection model will be deployed on an online detection platform. Thus, it must meet the requirements of high accuracy and speed. After comparing several common network models, we have chosen ResNet [16] as the backbone network for this study. The main reasons are as follows: First, ResNet, due to its simpler model structure, offers faster inference speed compared to other network models (such as VGG [17], Inception [18], or DenseNet [19]), which is crucial for real-time defect detection on the production line. Second, ResNet’s residual architecture is beneficial for learning defect features, and its simplicity makes it easier to fine-tune and optimize. Lastly, and most importantly, compared to other models, ResNet has relatively uniform operators, requiring less from the computation hardware, which makes it easier to deploy on FPGAs—a significant advantage in production environments with limited hardware resources. Within the ResNet series, we have selected ResNet18 because its number of layers is sufficient; more layers would result in longer recognition times. In addition, in the defect detection on the production line, we do not need to localize individual defect points, nor do we need to handle multiple defect points simultaneously. Therefore, the YOLO series [20] is not within our consideration. The ResNet18 model is divided into five stages. The first stage is a 7 × 7 convolution layer (Conv_1), and the following four stages are Conv2_x, Conv3_x, Conv4_x, and Conv5_x, each of which contains two residual blocks. The structure of the ResNet18 model is shown in Figure 2.

### 2.3. Feature Pyramid Network-Based Multiscale Feature Fusion Module

In the cigarette defect dataset, defects can occur at the interfaces, which are relatively small, or there might be larger contaminants or stains. Therefore, it is necessary to incorporate the feature pyramid network (FPN) [21] to provide a multi-scale representation of defect characteristics. FPN captures the correlations between shallow and deep features, thereby offering comprehensive multi-scale information. Additionally, as the patterns on the cigarette filter can bear similarities to certain defect features, leading to potential confusion, FPN’s top–down processing enables the propagation of deep semantic information back to shallow features, enhancing the semantic information of shallow features. This helps to improve the model’s ability to recognize defects with complex textures or subtle differences.

**Figure 3:**
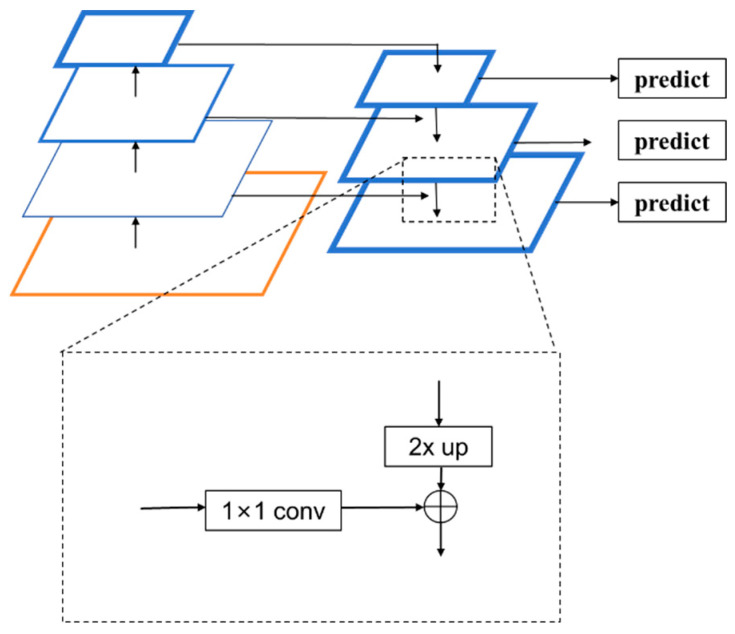
Structure of FPN.

The structure of the FPN is shown in Figure 3. As can be seen, the FPN comprises three main parts, i.e., bottom–up feature extraction, top–down upsampling, and lateral connections. With bottom–up feature extraction, the feature extraction network of the FPN is typically a regular convolutional network that extracts features from the input image. With each convolutional operation, the size of the feature map is halved, and its semantic level is elevated, ultimately forming a feature pyramid. This part of the network structure is commonly referred to as the downward path of feature extraction. In the top–down upsampling process, upsampling operations are performed on the top feature map, which increases the spatial resolution, thereby generating a new feature map with each enlargement. Before the lateral connections, the feature pyramid obtained from the first part undergoes feature fusion. A 1 × 1 convolution kernel is used to perform convolution on shallow features, which ensures consistency across channels for each layer. Then, the feature maps from the bottom–up and top–down pathways are connected at the same spatial scale, typically through an additive fusion process, such that the merged feature map contains both the high-resolution information of the lower-level features and the high-level semantic information of the upper-level features.

**Figure 4:**
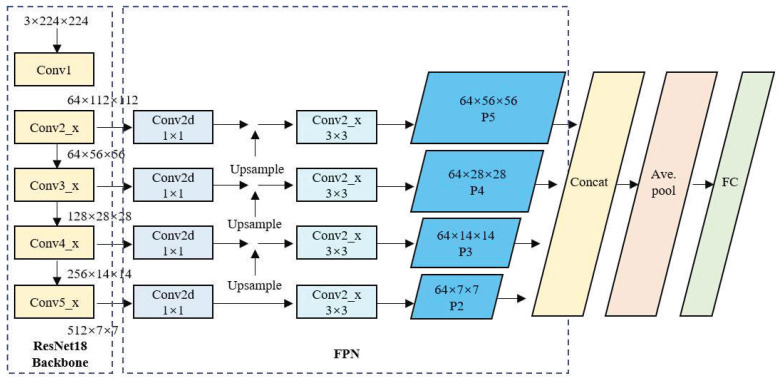
FPN-based ResNet18 network structure.

The structure of the FPN-based ResNet18 network is shown in Figure 4. Here, the input image data format is 3 × 224 × 224. Initially, ResNet18 performs the primary feature extraction through the C2, C3, C4, and C5 convolutional blocks. The sizes of the four extracted feature maps are 64 × 56 × 56, 128 × 28 × 28, 256 × 14 × 14, and 512 × 7× 7, respectively. These feature maps are fed laterally into the FPN, where 1 × 1 convolutions are performed to adjust the channel size to realize a consistent number of channels. Then, from top to bottom, upsampling is performed, and the upsampled feature maps are fused with the feature maps extracted from the preceding Conv block, which is then followed by a 3 × 3 convolution to obtain the final P2, P3, P4, and P5 feature maps of sizes 64 × 7 × 7, 64 × 14 × 14, 64 × 28 × 28, and 64 ×56 × 56, respectively. Then, for the smaller feature maps in the FPN, upsampling is performed again to increase their size to 64 × 56 × 56, which is the same as the largest feature map. Subsequently, a feature map cascading operation is implemented to obtain a feature map of size 256 × 56 × 56. Finally, the model proceeds through a fully-connected layer to produce the final five-class classification results.

### 2.4. Feature Enhancement and Attention Mechanism Modules

We have observed that the dataset collected from the production line exhibits a complex environment, with potential interference from tobacco debris, dust, and haze in the background. Additionally, the intricate patterns on cigarette filters can easily lead to confusion when detecting targets. To address these challenges, we have incorporated the convolution block attention module (CBAM) [22]. CBAM can select the most informative channel features for defect recognition through channel attention. And it can use spatial attention to highlight the spatial location of defects, thus enabling accurate defect localization against complex backgrounds. This coarse-to-fine approach of focusing attention mimics the human visual system’s method of filtering and processing information in complex scenes, effectively increasing the model’s ability to recognize defects within intricate patterns while reducing interference caused by the surrounding complex textures. By inserting the CBAM at the end of each convolutional block in the ResNet18 network, we enhance the focus on key feature maps. Consequently, the accuracy and robustness of the defect detection model are improved.

**Figure 5:**
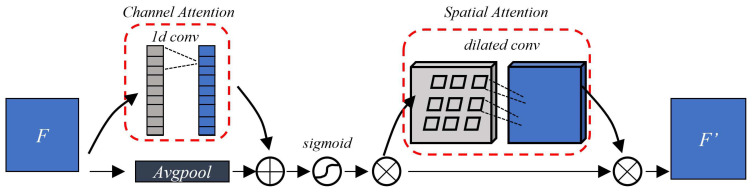
Structure of I-CBAM.

To minimize the additional hardware requirements of CBAM and enhance the algorithm’s hardware friendliness, we have designed an optimized version of CBAM, which we call I-CBAM. The common CBAM uses fully connected layers for computing channel attention to map features. However, the sequential computation inherent to fully connected layers cannot exploit data reuse effectively on FPGAs, resulting in a considerable wastage of computational resources. Additionally, within the spatial attention module, to aggregate a broader range of spatial contextual features, large 7 × 7 convolutional kernels are employed to assimilate spatial features. In contrast, most convolutions in ResNet use 3 × 3 kernels. The introduction of larger convolution kernels requires additional computational arrays and puts a strain on computational resources, significantly increasing the deployment difficulty when inserting traditional CBAM modules. Therefore, this paper proposes the use of 1d convolutions for channel attention to aggregate channel features, where the size of the 1d convolutional kernel corresponds to the number of channels in the neighborhood being aggregated. Due to the parameter-sharing property of the convolutional operations, computations are more hardware parallel compared to fully connected layers. For spatial attention, to maintain a consistent receptive field, this paper utilizes a 6 × 6 (four 3 × 3) dilated convolutions for aggregating 2d spatial attention features, thereby reducing the need for large convolution kernels. The structure of the I-CBAM is shown in Figure 5.

**Figure 6:**
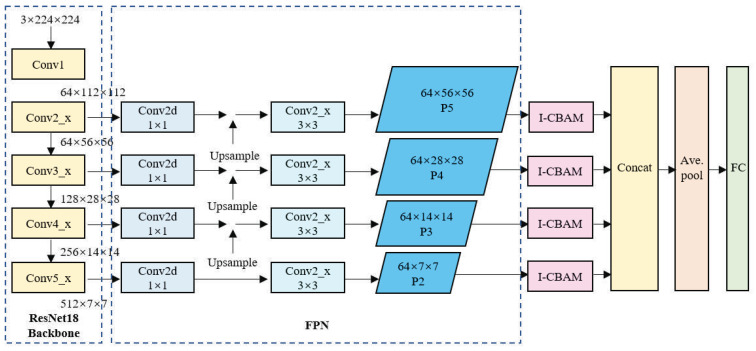
Structure of the proposed network.

The structure of the network with the feature enhancement module is shown in Figure 6. As shown, the network comprises three main components, i.e., the ResNet18 backbone for feature extraction, the FPN, and the I-CBAM. Here, the ResNet18 component retains only a series of convolutional layers and pooling layers from the conventional ResNet18 network, as well as some residual blocks with skip connections. This component is primarily responsible for extracting semantically informative features from the input image and producing feature maps of various scales. The FPN component combines low-resolution, semantically strong features with high-resolution, semantically weak features to enhance the feature maps obtained by the ResNet18 backbone. This is achieved through a top–down pathway, where the higher-level features are upsampled and merged with the lower-level features via lateral connections. Here, each merged feature map is processed by a 3 × 3 convolution operation to produce the final feature pyramid levels (P2, P3, P4, and P5). The I-CBAM component is designed to focus on information-rich features and suppress less useful features. Here, each feature map in the FPN passes through an I-CBAM block, which applies both channel and spatial attention to enhance the network’s feature expression ability in key areas.

After the operations of the I-CBAM module are completed, the sizes of the resulting feature map remain 64 × 7 × 7, 64 × 14 × 14, 64 × 28 × 28, and 64 × 56 × 56. Then, the feature maps are upsampled again to produce four feature maps of equal size, i.e., 64 × 56 × 56. Finally, the output image’s feature maps are concatenated to produce a feature map of size 256 × 64 × 64, and the final classification output is generated through a fully-connected layer.

### 2.5. Experiment and Result Analysis

In the process of training, we set the learning rate to 1 × 10^−4^, employed stochastic gradient descent as the optimizer, and used cross-entropy as the loss function to emphasize the correspondence between the predicted and the actual categories in the classification tasks. In addition, the batch size was set to 128 to balance the number of samples observed by the model before each weight update and the use of computational resources. The number of training epochs was set to 500 to ensure the model had sufficient opportunity to learn and optimize. To manage the learning rate dynamically and avoid getting stuck in local optima as much as possible during training, a learning rate reduction factor (Reduce_lr) of 0.3 was also established. ReLU was selected as the activation function due to its computational simplicity and effectiveness when handling nonlinear mapping issues in neural networks.

The performance of original ResNet18 for each defect type is shown in Table 1. Overall, the performance of the ResNet18 model on this dataset was not ideal. Specifically, the low F1-score value indicates that the model struggled to balance the false positive rates and detection rates. Upon further analysis of the performance across different categories, we observed some noticeable disparities. For example, the precision, recall, and F1-score for the “abnormal_align” category were relatively high, whereas the performance for the “abnormal_mix” category was comparatively poor. This disparity is largely attributed to the different number of samples for each category in the dataset. For the “abnormal_mix” category, there are fewer samples, and these defects also tend to be small and blend in with the background textures, thereby making them difficult to detect, which accounts for the poor performance. In contrast, the precision, recall, and F1-score results for the “normal” category are high. This is partly due to the larger number of samples with simpler features, thereby making it easier for the model to learn a limited representation of the features. In addition, anomalous samples are frequently more affected by noise interference, which can also reduce the model’s performance on such samples. In contrast, normal samples typically face less interference; thus, the model performs relatively better, thereby enhancing classification accuracy. These issues can impact the model’s performance, which can lead to variability in performance across different categories. To address these shortcomings, some improvement measures are required to enhance the detection precision and robustness of the model.

**Figure 7:**
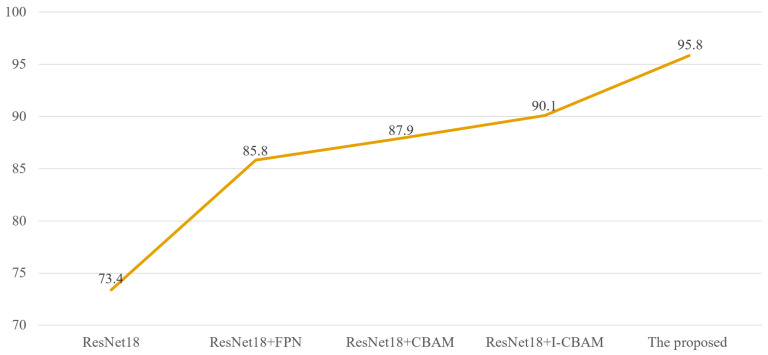
Comparison of accuracy indexes of each model (%).

Ablation studies were conducted to analyze and compare the performance of the ResNet18 model, ResNet18+FPN model, ResNet18+CBAM model, ResNet18+I-CBAM model, and the proposed model. The accuracy results for each model are shown in Figure 7. As can be seen, When the FPN was introduced into the model, the accuracy increased to 85.81%, representing a 12.41% improvement compared to the baseline model. Then, the CBAM was introduced by inserting it into the ResNet model’s convolutional blocks, which increased the model’s accuracy to 87.93%. Clearly, implementing the CBAM further optimized the important information in the feature maps and improved the accuracy by an additional 2.12% over the model with the FPN. These findings demonstrate that introducing attention mechanisms has a more significant impact on enhancing the model’s performance than the FPN when detecting small targets with complex defects. Subsequently, the CBAM was replaced with the improved I-CBAM, and we found that the accuracy increased to 90.12%, representing an improvement of 2.19% compared to using the CBAM. These results indicate that using the proposed CBAM can further improve the performance of the model. Finally, by adding both the FPN and the I-CBAM to the ResNet18 model, the model’s accuracy reached 95.88%, which is the highest of all the models, representing an improvement of 5.76% over the previous model and essentially satisfying the defect detection requirements of production lines.

When both the FPN and I-CBAM were integrated into the ResNet18 model, the advantages of both were leveraged fully. The FPN provides rich contextual information, which enables the model to understand the input data more comprehensively. In addition, the I-CBAM focuses on modeling the internal relationships within the model by using attention mechanisms to highlight certain key details. The combination of the two allows the model to perform exceptionally when processing information with complex internal structures, thereby achieving impressive predictive accuracy.

The experimental results for the precision metric are shown in Table 2. The overall trend is evident that the detection precision for all categories improved by adding the various feature enhancement modules. Comparing the five categories of cigarette defects, the precision for abnormal_align category improved from 77.82% in the baseline ResNet18 to 96.88% in the proposed model, an increase of nearly 19%. The precision for abnormal_fold category increased from 74.36% to 95.87%, representing an improvement of over 21%. For the abnormal_loose and abnormal_mix categories, ResNet18 had poor performance, but with the feature enhancement modules specifically designed for small targets with complex defects, the precision in the proposed model increased to 94.73% and 93.91%, with remarkable growth. The precision for the normal category achieved an higher level at 98.01%.

The experimental results for the recall metric are shown in Table 3. The proposed model consistently outperformed other models in all categories. Specifically, the abnormal_align category achieved the highest recall rate, indicating that abnormalities of this type were correctly detected and recognized by the model.

To demonstrate the impact of the added modules on hardware performance, we compared their parameter count, computational complexity, and actual running speed on a 3080Ti GPU. The result is shown in Table 4. which illustrates that the original ResNet18 model has fewer parameters and much faster running speed. However, after adding FPN and I-CBAM, although the speed is reduced by nearly 70%, the higher accuracy justifies this tradeoff, making it acceptable. Although I-CBAM appears to have a larger number of parameters than the regular CBAM and requires longer runtime on GPU platforms, for FPGAs, the operators in I-CBAM are much more friendly than those in CBAM, boasting better compatibility with the FPGA architecture.

To further evaluate the performance of different models on the target task, we performed training and comparative experiments on the CigDefect dataset with the MobileNetv3 [23], Inceptionv3 [18], YOLOv4 [24], YOLOv8s, YOLOv10s and proposed models. Here, approximately the same hyperparameter settings were used for all models during the training process to ensure fairness in the experiments. The experimental results are compared in Table 5. As can be seen, for lightweight networks like MobileNet, although they have fast processing speeds, their simple structures and fewer parameters make it difficult to extract complex and meaningful information. As for the YOLO series models, their advantages in multi-object detection cannot be fully utilized in this scenario and dataset. Additionally, their complex structures also slow down their speed. Therefore, in defect detection tasks involving small targets and complex textures, the design of feature fusion and attention mechanisms is crucial. Conventional image recognition algorithms often lose a significant amount of feature information, thereby failing to fully leverage these features. This further affects the recognition performance of the models.

## 3. Hardware Design Based on FPGA

### 3.1. Network Deployment Based on Xilinx DPU

For the complex texture defect detection task, a feature enhancement model based on the ResNet backbone network was designed and analyzed. However, Due to space and power constraints in certain special operating conditions, it is not suitable to use a large PC machine with a GPU as a terminal for detection. Therefore, we have designed a scheme to deploy and accelerate the model on an FPGA. As a reconfigurable computing platform, FPGAs offer strong parallel processing capabilities and high energy efficiency, thereby making them suitable to accelerate deep learning model inference tasks. By utilizing FPGA hardware resources effectively and designing specialized accelerators, we can enhance the computational efficiency of the proposed model and reduce energy consumption considerably, thereby allowing the model to be deployed on edge devices.

**Figure 8:**
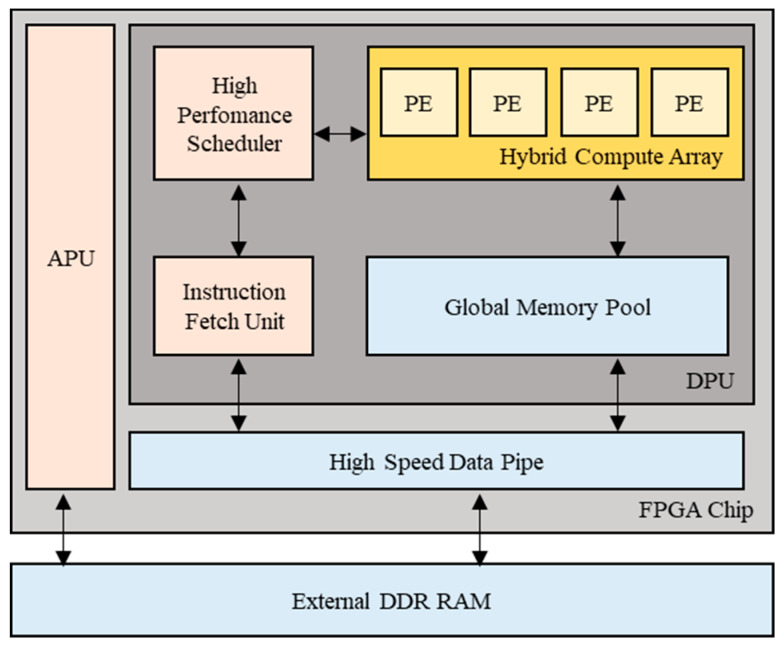
Block diagram of DPU application scheme.

Using Xilinx’s Vitis AI development platform, we analyze and optimize the model, and we customize an efficient DPU acceleration engine based on its computational characteristics. By configuring the DPU and mapping strategies, we further optimize the overall performance of the system. Finally, we evaluate the resource consumption and performance of the accelerator under different architectures and different numbers of acceleration cores, and the results validate the rationality and superior performance of this accelerator design. The DPU used in these experiments was the DPUCZDX8G [25], which was specifically designed for the Zynq UltraScale+ MPSoC. The application scheme of the DPUCZDX8G is shown in Figure 8. This device reads instructions from external storage at startup to control the operation of the computing engine through the instruction scheduler, which completes various tasks, e.g., fetching, decoding, and dispatching. The data read from the data port are transmitted from the external storage through the on-chip buffer to the computing engine for computation of processing elements (PE), and the results computed by the PEs are also written out through the data port. The on-chip buffer controller is designed to cache input, intermediate, and output data to achieve high throughput and efficiency.

**Figure 9:**
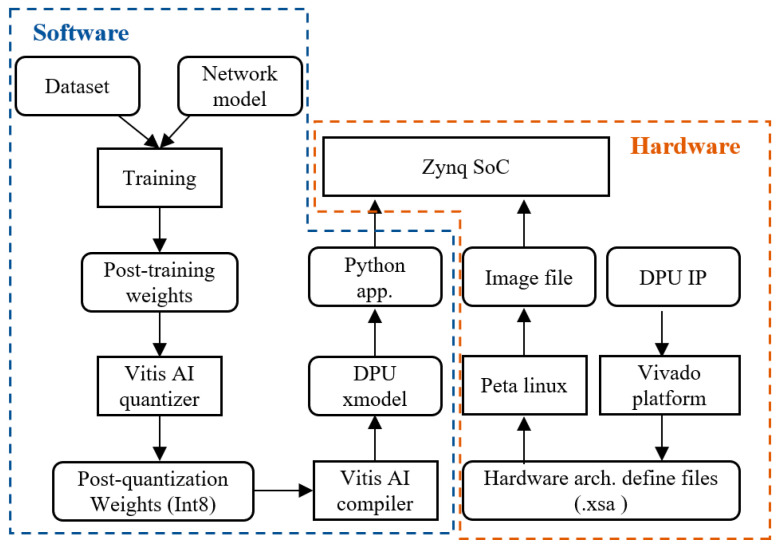
Network deployment flow on DPU.

Figure 9 shows the full process of deploying our model on the DPU. Initially, the trained model is input to the Vitis AI quantizer, where it undergoes quantization from a 32-bit floating-point to an 8-bit fixed-point model, thereby reducing the size of the model and improving its operational efficiency. The next phase is the model compilation stage, where the Vitis AI compiler compiles the quantized model into an xmodel file that can run efficiently on the DPU. The compilation process involves model optimization, instruction conversion, and other operations. Here, the algorithms are converted to DPU instructions to fully utilize the DPU’s computational capabilities. The final phase is model deployment, where the compiled xmodel file is stored in the operating system using an image file to boot the Linux system on the process system (PS) side, and the application program on the PS side is executed to call the xmodel to complete the model’s inference tasks.

To further improve the detection speed, we tried a dual-core DPU parallel scheme, albeit at the cost of sacrificing some hardware resources and power consumption on the FPGA. We conducted tests on a dataset consisting of 2000 test images with an input size of 224 × 224. After preprocessing, the images were input to the DPU for inference, and the measured inference latency is shown in Table 6. As can be seen, the inference speed of the dual-core DPU improves by more than 1.5 times compared to the single-core DPU. Thus, when resources are sufficient, selecting a dual-core DPU has a significant advantage in terms of inference speed.

### 3.2. Detection System Design

**Figure 10:**
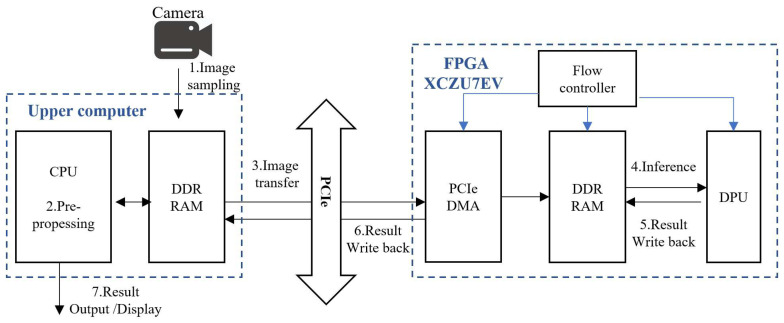
Application system architecture.

The application system diagram of the defect detection system is shown in Figure 10. As shown, the system is divided into two main parts, i.e., the host computer and the FPGA, which complete data transmission through PCIe DMA. In Figure 10, the blue arrows represent the control flow, and the black arrows represent the data flow.

First, the host computer captures cigarette images using the camera device and stores them in a designated location. Then, the host computer reads the image data from the specified location and proceeds to preprocess the images, including cropping, scaling, and mean normalization operations. After preprocessing, the processed image data and the written control signal are transferred together to the FPGA’s DDR memory via PCIe. On the FPGA side, the flow controller reads the written control signal, and then the DPU retrieves the input data and model parameters from DDR memory and begins to execute the inference task. During the inference process, the DPU writes the inference results and progress signals back to the DDR memory. Subsequently, the inference results are sent back to the host computer via PCIe for direct output, and the progress signals are passed to the host computer’s PCIe controller to continue the next data transmission.

The PCIe controller on the host computer side (based on the control signal from the FPGA side and its own control logic) completes the transmission of the input data and written control signals. After the flow controller on the FPGA side reads the written control signals from the DDR memory, it controls the DPU to read the input data and model parameters from DDR memory. The DPU initiates a call request, invoking related library functions to complete the inference task. Simultaneously, the flow controller reads the progress control signals written by the DPU from DDR memory and controls the PCIe controller on the FPGA side to send back the inference results and progress signals. After the PCIe on the FPGA side recognizes the request from the flow controller, it completes the data return task through PCIe.

It is noteworthy that the upper computer does not necessarily have to be a PC, nor is the system’s architecture required to be as Figure 10. This FPGA-based detection system can operate independently of other devices, or it can be connected to other industrial control equipment. It can even directly connect the camera to the FPGA to function as an integrated whole for the purpose of detection. This is made possible thanks to the rich array of interfaces and the exceptionally high flexibility of FPGAs, as well as their superior power efficiency.

### 3.3. Dual Buffer Scheme

**Figure 11:**
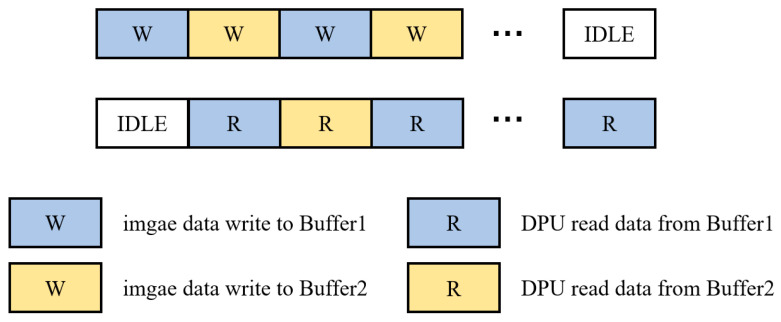
Dual buffer operation between computer and DPU.

In the defect detection system, the data are preprocessed before being transmitted to the FPGA for inference via PCIe. However, if the data transmission and inference tasks are executed in series, the efficiency would be very low because, in serial mode, the FPGA must wait for the data transmission to complete before beginning the inference task, and only after the inference task is finished can the data transmission of the next frame of images begin. This results in a significant amount of idle time between the data transmission and inference tasks. Thus, the system’s hardware resources are not utilized fully, and the overall efficiency of the system is reduced. To address this issue, we adopted a dual buffer design. Dual buffering is commonly used to optimize data transmission and processing, with the basic principle of using two buffers to alternate the data reading and writing processes. When one buffer is being written with data, the other buffer can be read data simultaneously. As a result, data transmission and processing can occur in parallel; thus, read/write conflicts are avoided, and the data throughput is increased. Figure 11 shows the dual buffer design implemented in the system.

### 3.4. Implementation Results

**Figure 12:**
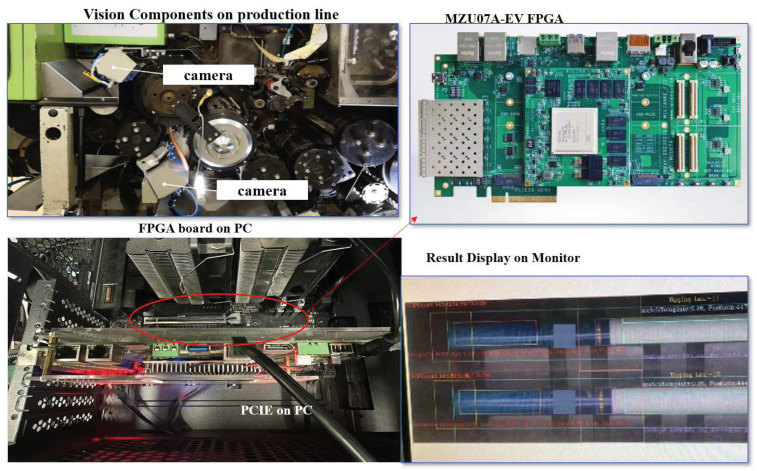
Implementation of the defect detection hardware system.

Figure 12 shows the implementation of the defect detection hardware system. As can be seen, the FPGA is connected to the PC motherboard via the PCIe interface. And the PC is connected to the vision component system on a production line. The FPGA side application runs on the Linux system on the FPGA side, including data reading, invoking the DPU, DPU inference, control logic, and the output and writing back of the results. The host computer application runs on the PC side, which includes image reading, image preprocessing, image transfer, and control logic. The detected results are shown on a screen.

**Figure 13:**
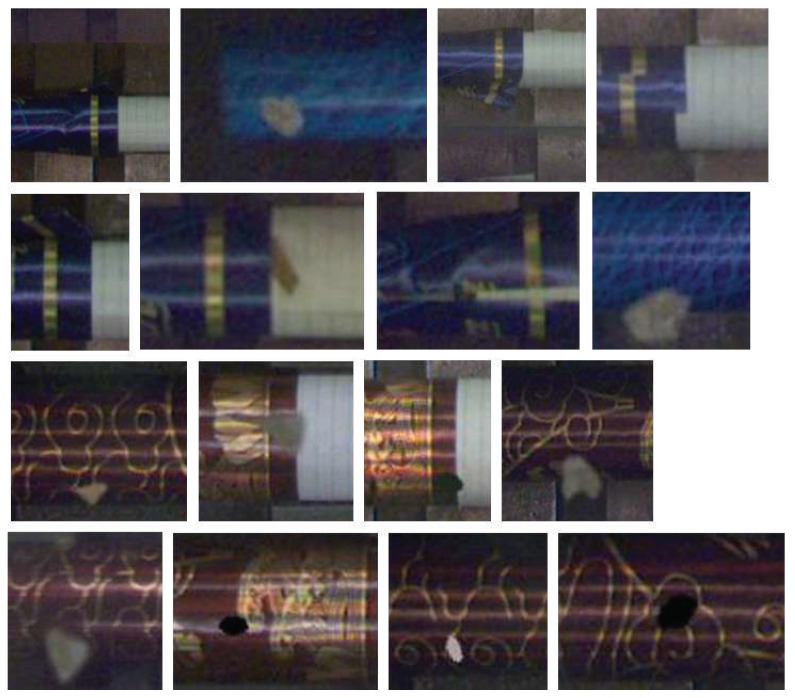
The defect patterns detected by the proposed hardware system.

The defects detected by the proposed hardware system are shown in Figure 13. Tests were performed on two types of products: red and blue. The pattern on the red product is slightly simpler than on the blue one, but its color is closer to that of the production line background. As seen, all types of defects can be successfully identified. Unlike YOLO serials, which have object detection and segmentation capabilities, this model does not offer bounding-box detection. Instead, the results are sent to the supervisory control system in the form of data packets, which are used to control the rejection of defective products.

In addition to the DPU, the PCIe interface IP for data transfer, related control IP cores, and routing resources were also introduced to construct a complete system. The comprehensive hardware resource consumption of the obtained system is shown in Table 7.

Table 8 shows the statistics for the time consumption of various tasks during the system’s operation, including the average total time required to complete testing of the 2000 images with an input size of 1100 × 320. Here, the term “latency” refers to the end-to-end delay, which is the total time consumed from reading an image to obtaining the corresponding final result. Through testing, without the dual buffer scheme, the system’s average end-to-end latency per image was 12.80 ms. However, after incorporating the dual buffer scheme, the system’s average end-to-end latency per image was reduced to only 9.382 ms, translating to a real-time processing speed of 106.6 FPS.

A comparison of the latency of the proposed system and other platforms is shown in Table 9. In separate inference tasks, the proposed FPGA implementation significantly reduced the inference latency from 45.59 ms to 3.843 ms compared to the Intel Xeon Silver 4210R CPU (Intel, Santa Clara, CA, USA), achieving nearly a 12-fold improvement in speed. Compared to the Nvidia 3080Ti GPU (Nvidia, Santa Clara, CA, USA), the inference speed of the proposed FPGA implementation was improved by nearly two times. Comparisons of the end-to-end latency also confirm the superior performance of the proposed system. Compared with the solution using only the Intel Xeon CPU, the end-to-end latency was reduced from 50.06 ms to 9.382 ms, representing a more than fivefold speed increase. In addition, there is also a significant reduction in end-to-end latency compared to the combination solution with the Nvidia 3080Ti GPU. These results underscore the importance and applicability of the proposed system in scenarios requiring real-time performance. Overall, the proposed system can complete the same tasks in less time, which is important for applications with real-time requirements.

To evaluate our FPGA inference accelerator thoroughly, Table 10 shows an extensive comparison of energy efficiency between the proposed system and several cutting-edge implementations. The results demonstrate that the Xilinx MZU07A-EV (Xilinx, San Jose, CA, USA) FPGA platform utilized in the current study achieved an impressive throughput of 702.5 GOPS at INT8 precision while running the proposed model, thereby outperforming similar efforts. In addition, this platform boasts a minimal power consumption of only 14.42 W, resulting in an unparalleled energy efficiency ratio of 48.72 GOPS/W, thereby marking it as the most efficient among the compared systems.

In terms of throughput, our design outperformed similar Xilinx FPGA platforms, e.g., the VX980T and XCVU9P, which deliver throughputs of 600 GOPS and 484.21 GOPS, respectively. The proposed accelerator system also outperformed the high-performance 3080Ti GPU in terms of both throughput and energy efficiency, with the latter’s energy efficiency ratio of 4.392 GOPS/W significantly falling short of the MZU07A-EV FPGA platform’s performance. These observations underscore the remarkable energy efficiency improvements provided by FPGAs. In addition, the PYNQ-Z1 benefits from low power consumption at 1.44 W; however, it achieves a throughput of only 63.3 GOPS and an energy efficiency ratio of 43.9 GOPS/W, which illustrates the compromise between power and performance. In demanding deep learning tasks, the proposed FPGA solution achieves an optimal balance between power consumption and throughput, thereby offering a highly efficient approach. From an energy efficiency perspective, the proposed FPGA scheme stands out in terms of power consumption and exhibits substantial potential in terms of operations per watt.

## 4. Discussion

In this study, we initiated an exploration into the feasibility of using enhanced convolutional neural networks for the specific task of cigarette filter defect detection in real-world manufacturing environments. By comparing the performance of various architectures such as a standard ResNet18 against versions augmented with feature enhancement techniques like FPN and CBAM, we gained several insights that are crucial for selecting appropriate models for deployment in industrial settings.

We observed that the basic ResNet18 provided a solid foundation due to its robust feature extraction capabilities. However, incorporating feature enhancement techniques such as FPN and CBAM resulted in a marked increase in accuracy. These enhancements enabled the models to have a better detection capability, especially for small defects and intricate patterns that are common in high-quality cigarette filters. The integration of FPN proved to significantly bolster the model’s ability to integrate semantic information from deep layers with low-level features, addressing the challenges of defect detection on complex patterns. CBAM, on the other hand, allowed the model to focus on important features selectively, thereby further improving the accuracy.

A notable discovery was that after taking into consideration the compatibility of both software and hardware, feature-enhanced models did not substantially compromise inference speed, which is critical for on-the-fly defect detection in manufacturing lines. This suggests that a well-designed, feature-enhanced CNN can meet the dual requirements of high accuracy and high speed in industrial applications.

In the manufacturing sector, especially within the high-volume production lines, defect detection models must achieve a delicate balance between accuracy and speed. Standards typically demand accuracies upwards of 90%, and processing times are short enough to evaluate each item on the assembly line in real time. Our enhanced ResNet18 model achieved an accuracy of 95.88%, surpassing the standard threshold, while maintaining an impressive end-to-end processing speed of 9.38 ms per image frame, well within the bounds for real-time analysis. Our model not only meets the stringent accuracy requirements but does so with a speed sufficient for inline defect detection. This dual achievement is often difficult due to the trade-offs between complexity and speed in neural network models. Hence, the proposed methods and models exceed the requisite standards, making them novel and immensely practical for deployment. The effectiveness of the FPGA-accelerated deployment model indicates that the solution is not just theoretically sound but also practically applicable. Given the nature of defect detection, where manufacturing lines cannot afford to stop for batch processing, the ability to process each item in real time with high accuracy equates to significant improvements in quality control and reduction in waste.

In conclusion, the insights derived from our comparisons have revealed that models like ResNet18 can be significantly improved without compromising on industrial applicability. The enhanced models meet and exceed typical industrial standards, indicating a promising direction for future research and real-world applications. By balancing computational efficiency with accurate detection ability, we provide a blueprint for state-of-the-art defect detection systems that can be readily integrated into current production lines. This not only enhances the quality of the end product but also exemplifies the practical application of AI in modern manufacturing.

## 5. Conclusions

The high-speed detection of complex patterned cigarette filter defects on a production line is an engineering challenge. To address this issue, this paper proposes a neural network model based on the ResNet18 model that incorporates a feature enhancement module and attention mechanism. Through experimental evaluation and comparison, the results demonstrate that the model achieves a detection accuracy of 95.88% on a dataset of cigarette filter defects. Subsequently, the proposed model is deployed on an FPGA, and a system is constructed to integrate it into real-world operating conditions. The results show that the test system achieves a detection speed of 106.6 FPS with a frame image delay of 9.38 ms, meeting the real-time requirements of the production line.

## Figures and Tables

**Table 1:** ResNet18 performance (%).

Defect Type	Precision	Recall	F1-Score
abnormal_align	77.82	75.37	74.04
abnormal_fold	74.36	73.59	73.01
abnormal_loose	68.45	63.24	65.03
abnormal_mix	65.72	58.93	61.02
normal	78.13	76.48	77.05

**Table 2:** Precision of each defect type (%).

Type	ResNet18	ResNet18 + FPN	ResNet18 + CBAM	ResNet18 + I-CBAM	Proposed Network
abnormal_align	77.82	86.53	88.67	90.99	**96.88**
abnormal_fold	74.36	85.78	87.94	90.15	**95.87**
abnormal_loose	68.45	84.72	86.88	88.89	**94.73**
abnormal_mix	65.72	84.35	86.22	88.43	**93.91**
normal	78.13	87.67	89.93	92.13	**98.01**

**Table 3:** Recall of each defect type (%).

Type	ResNet18	ResNet18 + FPN	ResNet18 + CBAM	ResNet18 + I-CBAM	Proposed Network
abnormal_align	75.37	86.10	88.02	90.41	**96.30**
abnormal_fold	73.59	83.97	86.61	89.28	**94.73**
abnormal_loose	63.24	83.59	85.41	87.61	**93.86**
abnormal_mix	58.93	82.98	84.73	87.06	**92.53**
normal	76.48	86.23	88.47	91.65	**97.29**

**Table 4:** Model size and GFLOPs of each model.

Algrithms	ResNet18	ResNet18 + FPN	ResNet18 + CBAM	ResNet18 + I-CBAM	Proposed Network
Parameters(M)	11.2	11.4	18.8	20.4	**20.6**
GFLOPs	4.69	5.55	10.20	11.55	**12.40**
FPS on 3080Ti	465.35	160.98	151.20	142.11	**133.39**

**Table 5:** Performance comparison of five networks (%).

Perfomance	MobileNetv3	Inceptionv3	YOLOv4	YOLOv8s	YOLOv10s	Proposed Network
Precision	70.51	78.03	84.03	80.80	76.25	**95.88**
Recall	67.02	76.80	80.11	82.50	81.67	**94.94**
Parameters	5.40	21.10	63.40	11.10	8.20	**20.60**
GFLOPs	1.40	25.90	81.90	28.40	20.80	**12.40**
FPS on 3080Ti	568.71	75.60	32.50	70.36	82.40	**133.39**

**Table 6:** Single and dual DPU hardware resource usage and inference latency.

Resource of FPGA	Total in XCZU7EV	Single B4096 DPU Utilization	Dual B4096 DPU Utilization
LUT	230,400	58,684 (25.47%)	107,781 (46.78%)
LUTRAM	101,760	6131 (6.02%)	11,731 (11.53%)
FF	460,800	106,199 (23.05%)	204,313 (44.34%)
BRAM	312	111 (35.58%)	218 (69.87%)
URAM	96	40 (41.67%)	80 (83.33%)
DSP	1728	704 (40.74%)	1394 (80.67%)
Inference latency	/	6.200 ms	3.843 ms

**Table 7:** FPGA hardware resources.

Resource Type	DPU Occupied	Total Occupied	Utilization
LUT	123,873	230,400	53.76%
LUTRAM	14,121	101,760	13.88%
FF	255,195	460,800	55 38%
BRAM	250	312	80.13%
URAM	80	96	83.33%
DSP	1,394	1,728	80.67%

**Table 8:** Latency of each task (ms).

Task	Latency
Load images	2.088
Preprocessing	2.117
Inference in DPU	3.843
Data transfer	2.412
Total latency without dual buffer	12.80
Total latency with dual buffer	9.382

**Table 9:** Latency comparison between different platforms (ms).

Platform	Inference Latency	End-to-End Latency
Nvidia 3080Ti GPU+Intel Xeon Sliver 4210R	7.497	13.81
Intel Xeon Silver 4210R CPU	45.59	50.06
Proposed system	3.843	9.382

**Table 10:** Comparison of energy efficiency comparison of different platforms.

Method	Model	Precision	Platform	Performance (GOPS)	Power (W)	Energy Efficiency (GOPS/W)
Ref [26]	ResNet20	FP16	PYNQ-Z1	63.3	1.440	43.9
Ref [27]	ResNet101	FP16	VX980T	600	12.39	48.43
Ref [28]	ResNet18	FP16+FP32	XCVU9P	484.21	22.38	21.64
Ref [29]	ResNet18	INT8	XC706	124.9	7.31	17.09
This work	**proposed model**	**INT8**	**3080Ti GPU**	**360.1**	**82**	**4.392**
	**proposed model**	**INT8**	**MZU07A-EV**	**702.5**	**14.42**	**48.72**

## Data Availability

The dataset involved in this article is currently under an ongoing study and is therefore not available in a publicly accessible repository. If you need to obtain the dataset, please contact the authors.

## References

[1] Cheng X., Yu J. (2020). RetinaNet with difference channel attention and adaptively spatial feature fusion for steel surface defect detection. IEEE Trans. Instrum. Meas..

[2] Aghababaeyan Z., Abdellatif M., Briand L., Ramesh S., Bagherzadeh M. (2023). Black-box testing of deep neural networks through test case diversity. IEEE Trans. Softw. Eng..

[3] Yang H., Chen Y., Song K., Yin Z. (2019). Multiscale feature-clustering-based fully convolutional autoencoder for fast accurate visual inspection of texture surface defects. IEEE Trans. Autom. Sci. Eng..

[4] Zhang J.J., Gu T., Basu K., Garg S. Analyzing and mitigating the impact of permanent faults on a systolic array based neural network accelerator. Proceedings of the 2018 IEEE 36th VLSI Test Symposium (VTS).

[5] Kumaresan S., Aultrin K.J., Kumar S., Anand M.D. (2021). Transfer learning with CNN for classification of weld defect. IEEE Access.

[6] Wei X., Yang Z., Liu Y., Wei D., Jia L., Li Y. (2019). Railway track fastener defect detection based on image processing and deep learning techniques: A comparative study. Eng. Appl. Artif. Intell..

[7] Baygin M., Karakose M., Sarimaden A., Erhan A. Machine vision based defect detection approach using image processing. Proceedings of the 2017 International Artificial Intelligence and Data Processing Symposium (IDAP).

[8] Lin C.H., Ho C.W., Hu G.H., Kuo P.C., Hu C.Y. Alloy Cast Product Defect Detection Based on Object Detection. Proceedings of the 2021 International Symposium on Intelligent Signal Processing and Communication Systems (ISPACS).

[9] Lien P.C., Zhao Q. Product surface defect detection based on deep learning. Proceedings of the 2018 IEEE 16th Intl Conf on Dependable, Autonomic and Secure Computing, 16th Intl Conf on Pervasive Intelligence and Computing, 4th Intl Conf on Big Data Intelligence and Computing and Cyber Science and Technology Congress (DASC/PiCom/DataCom/CyberSciTech).

[10] Krizhevsky A., Sutskever I., Hinton G.E. (2012). Imagenet classification with deep convolutional neural networks. Adv. Neural Inf. Process. Syst..

[11] Liu H., Yuan G., Yang L., Liu K., Zhou H. (2022). An appearance defect detection method for cigarettes based on C-CenterNet. Electronics.

[12] Qu H., Zhang P., Zhang K., Wu J. (2017). Research on cigarette filter rod counting system based on machine vision. Proceedings of the Advanced Computational Methods in Life System Modeling and Simulation: International Conference on Life System Modeling and Simulation, LSMS 2017 and International Conference on Intelligent Computing for Sustainable Energy and Environment, ICSEE 2017.

[13] Sheng F., Song S., Xia S. A real-time cigarettes counting and loose ends detection algorithm. Proceedings of the 2016 IEEE International Conference of Online Analysis and Computing Science (ICOACS).

[14] Cao J.L., Li J.F., Da Lu T. (2016). A Cigarette Surface Defect Detection System Based on Data Acquisition Card. MATEC Web Conf..

[15] Xiao Z. (2018). Research and Implementation of Cigarette Defect Detection Algorithm.

[16] Targ S., Almeida D., Lyman K. (2016). Resnet in resnet: Generalizing residual architectures. arXiv.

[17] Vedaldi A., Zisserman A. (2016). Vgg Convolutional Neural Networks Practical.

[18] Szegedy C., Vanhoucke V., Ioffe S., Shlens J., Wojna Z. Rethinking the inception architecture for computer vision. Proceedings of the IEEE Conference on Computer Vision and Pattern Recognition.

[19] Zhu Y., Newsam S. Densenet for dense flow. Proceedings of the 2017 IEEE International Conference on Image Processing (ICIP).

[20] Jiang P., Ergu D., Liu F., Cai Y., Ma B. (2022). A Review of Yolo algorithm developments. Procedia Comput. Sci..

[21] Lin T.Y., Dollár P., Girshick R., He K., Hariharan B., Belongie S. Feature pyramid networks for object detection. Proceedings of the IEEE Conference on Computer Vision and Pattern Recognition.

[22] Woo S., Park J., Lee J.Y., Kweon I.S. Cbam: Convolutional block attention module. Proceedings of the European Conference on Computer Vision (ECCV).

[23] Howard A., Sandler M., Chu G., Chen L.C., Chen B., Tan M., Wang W., Zhu Y., Pang R., Vasudevan V. Searching for mobilenetv3. Proceedings of the IEEE/CVF International Conference on Computer Vision.

[24] Bochkovskiy A., Wang C.Y., Liao H.Y.M. (2020). Yolov4: Optimal speed and accuracy of object detection. arXiv.

[25] XDC DPUCZDX8G for Zynq UltraScale+ MPSoCs Product Guide (PG338). https://www.xilinx.com/support/documentation/ip_documentation/dpu/v33/pg338-dpu.pdf.

[26] Kim V.H., Choi K.K. (2023). A reconfigurable CNN-based accelerator design for fast and energy-efficient object detection system on mobile FPGA. IEEE Access.

[27] Huang W., Wu H., Chen Q., Luo C., Zeng S., Li T., Huang Y. (2021). FPGA-based high-throughput CNN hardware accelerator with high computing resource utilization ratio. IEEE Trans. Neural Netw. Learn. Syst..

[28] Fang C., Sun W., Zhou A., Wang Z. (2023). Efficient N: M Sparse DNN Training Using Algorithm, Architecture, and Dataflow Co-Design. IEEE Trans. Comput. Aided Des. Integr. Circuits Syst..

[29] Xiao Q., Liang Y. Zac: Towards automatic optimization and deployment of quantized deep neural networks on embedded devices. Proceedings of the 2019 IEEE/ACM International Conference on Computer-Aided Design (ICCAD).

